# A functional *FRIa* variant drives turnip adaptation to the Qinghai-Xizang Plateau

**DOI:** 10.1186/s43897-025-00185-9

**Published:** 2026-01-12

**Authors:** Yan Zheng, Danni Yang, Landi Luo, Xin Yin, Xingyu Yang, Yunqiang Yang, Xiangxiang Kong, Yongping Yang

**Affiliations:** 1 CAS Key Laboratory of Tropical Plant Resources and Sustainable Use, Xishuangbanna Tropical Botanical Garden, Chinese Academy of Sciences, Menglun, 666303 MenglaYunnan China; 2 Germplasm Bank of Wild Species, Yunnan Key Laboratory for Crop Wild Relatives Omics, Kunming Institute of Botany, Chinese Academy of Sciences, Kunming, 650201 Yunnan China; 3 University of Chinese Academy of Sciences, Beijing, 100049 China

**Keywords:** Turnip, Flowering time, *FRIa* variation, Adaptation, Qinghai-Xizang Plateau

## Abstract

Flowering time is crucial for plant adaptation to regional climate conditions and for reproduction in agricultural production. Turnip (*Brassica rapa* ssp. *rapa*) is a traditional adaptive vegetable cultivated on the Qinghai-Xizang Plateau. However, little is known about the potential molecular mechanisms underlying the adaptive evolution of flowering time in turnip in different regions, especially on the Qinghai-Xizang Plateau. In this study, the key adaptive trait of flowering time, which is positively correlated with altitude, was analyzed in 104 turnip accessions grown worldwide. Whole-genome resequencing and Sanger sequencing discovered that the *FRIa* gene, a key regulator of flowering time in turnip, had sequence variations, with five distinct alleles and eight genotypes being defined. By combining the association analyses of *FRIa* genotypes and flowering time as well as genetic validation, *FRIa1* was identified as a highly functional allele that contributes to delayed flowering of turnip for adaptation to the Qinghai-Xizang Plateau. Four specific SNP variations were determined to be critical for the strong function of *FRIa* in turnip. Our results provide new insights into the adaptation of flowering time in turnip along altitudinal gradients and shed light on the adaptive evolution of *FRIa* in turnip on the Qinghai-Xizang Plateau.

**Supplementary Information:**

The online version contains supplementary material available at 10.1186/s43897-025-00185-9.

## Core

*FRIa* variation plays a key role in the adaptive trait of flowering time in turnip. High-altitude *FRIa *alleles in turnip are described as highly functional alleles that contribute to late flowering of turnip during adaptation to the Qinghai-Xizang Plateau. Four specific SNP variations in *FRIa* in turnip were identified to be critical for its function in delaying flowering time.

## Gene & accession numbers

*FRIa* (BrrGene0096000), *FLC2* (BrrGene0414960), *SVP* (BrrGene0297860),* MAF1* (BrrGene0414960),* MAF2* (BrrGene0176850),* MAF3* (BrrGene0547080),* MAF4* (BrrGene0172410),* MAF5* (BrrGene0502720). Sequence data of* FRIa* alleles can be found in the Gene Bank databases with the following accession numbers:* FRIa1* accession: PQ067249, *FRIa2* accession: PQ067250, *FRIa3* accession: PQ067251, *FRIa4 *accession: PQ067251, *FRIa5 *accession: PQ067252.

## Introduction

Flowering time is crucial for the reproductive success of plants and significantly impacts crop yield and quality in agricultural production. Furthermore, it is key to plant adaptation to various environmental conditions (Mitchell-Olds and Schmitt 2006). As crop varieties spread to different environmental conditions, flowering time is subject to strong selective pressures, especially in annual crops, enabling the adaptation of plants to new environments (Fitter and Fitter 2002; Craufurd and Wheeler 2009; Wilczek et al. 2010). Local adaptation along latitudinal, altitudinal, and climatic gradients is strongly associated with the potential genetic variation and adaptive evolution within species (Böcher 1949; Schlenker and Roberts, 2009; Méndez-Vigo et al. 2011). Changes in flowering time significantly influence the breeding of *Brassica* vegetables to ensure adaptation to local climate conditions. Turnip (*Brassica rapa* subsp. *rapa*), a *Brassica* vegetable, is one of the oldest root vegetables and is thought to be originated in the Mediterranean region (Qi et al. 2017). As an ancient cultivated species, the domestication of the turnip by humans has been a long process in which natural selection plays a significant role (Purugganan et al., 2019). Therefore, a better understanding of adaptive changes in flowering time in turnip can facilitate the cultivation of new varieties that are better suited to their planting environments with improved agricultural traits.

Natural genetic variations in two key flowering genes, *FRIGIDA* (*FRI*) and *FLOWERING LOCUS C* (*FLC*), have been shown to be major determinants of the variations in flowering time in *Arabidopsis* (Johanson et al. 2000; Caicedo et al. 2004; Shindo et al. 2005). In winter-annual *Arabidopsis*, FRI plays a key role in promoting the transcription of the strong floral repressor *FLC* to inhibit flowering before vernalization (Shindo et al. 2005). As a scaffold protein, FRI forms a transcriptional activation complex with FRGIDA LIKE 1 (FRL1), SUPPRESSOR OF FRIGIDA 4 (SUF4), FRIGIDA ESSENTIAL 1 (FES1) and FLC EXPRESSOR (FLX), which further interacts with the H3K4 methyltransferase complex COMPASS-like, histone acetyltransferases, SWR1 chromatin removing complex (SWR1-C) and other chromatin modifiers to form a super-complex that enriches around the transcription start site of *FLC*, thereby promoting high-level expression of *FLC* (Choi et al. 2011; Li et al. 2018). Although natural variation in *FLC* has been identified, it occurs relatively infrequently. Variation in flowering time is explained primarily by allelic variation in *FRI* and its epistatic interaction with *FLC* (Caicedo et al. 2004; Lempe et al. 2005; Zhang and Jiménez-Gómez 2020). However, little is known about the key genetic variations affecting flowering time in species other than *Arabidopsis*. Studies have investigated flowering time variation in some *A. thaliana* relatives, such as *Arabidopsis lyrata* (Leinonen et al. 2013), *Brassica napus* (Raman et al. 2016), and *Capsella rubella* (Yang et al., 2018).

In *Brassica* species, due to whole genome triplication, there are two *FRI* homologues (*FRIa* and *FRIb*) and four *FLC* homologues (*FLC1*, *FLC2*, *FLC3*, and *FLC5*) in diploid species *B. rapa* and *B. oleracea* (Fadina and Khavkin 2014; Akter et al. 2021). In addition to FLC5, which is characterized as a weak flowering regulator (Xi et al. 2018), FLC1, FLC2, and FLC3 have been reported to function as floral repressors (Akter et al. 2021). Although *FLC1* and *FLC3* have been associated with flowering time in *B*. *rapa* (Kakizaki et al., 2011; Kitamoto et al. 2014), multiple quantitative trait locus (QTL) analyses across *Brassica* species, including *B*. *rapa*, *B. oleracea* and *B. napus*—have consistently identified *FLC2* as a key floral repressor (Zhao et al. 2010; Okazaki et al. 2007; Wu et al. 2012; Xiao et al. 2013; Ridge et al., 2015; Raman et al. 2016; Tudor et al., 2020). In *Arabidopsis*, FRI is the primary determinant of natural variation in flowering time (Johanson et al. 2000; Li et al. 2018). In *Brassica* species, compared with *FRIb*, sequence variation and functional analysis of *FRIa* have demonstrated its significant role in regulating flowering time in *B. rapa*, *B. oleracea* and *B. napus* (Wang et al., 2011; Irwin et al. 2012; Yi et al., 2018; Zheng et al., 2021). These findings highlight the pivotal role of *FLC2* and *FRIa* in determining flowering time variation in *Brassica* crops.

Turnip is widely cultivated in Europe, Central Asia, South Asia, and East Asia and is one of the few cultivated crops that can adapt to the climate conditions of the Qinghai-Xizang Plateau (Zheng et al. 2023). Investigating the molecular basis of flowering time variation in turnip will expand our understanding of the genetic factors that contribute to the adaptation of flowering time and the formation of local varieties. In this study, to investigate the variation of flowering time and the underlying genetic mechanisms among different accessions of turnip, 104 local accessions from around the world were collected for flowering time analysis, which is the most extensive collection of turnip accessions for analyzing flowering time to date. The flowering time of turnip from different geographical regions showed significant variations, which is closely related to the altitude and climate temperature of their growing regions. As the major genetic determinant of variations in flowering time, the genetic variations of the homologue of *FRI* and *FLC* and their contribution to differences in flowering time were further investigated. The results revealed the key role of *FRIa* variations in maintaining the late flowering time of high-altitude turnips, as well as the crucial role of variations in key single nucleotide polymorphisms (SNPs) in the evolution of highly functional *FRIa*. Our results provide new insights into the variation in *FRIa*, facilitating timely flowering in turnip under different altitudinal conditions, which reveals novel evolution of *FRIa* alleles on the Qinghai-Xizang Plateau.

## Results

### Ecogeographic adaptation of turnip with respect to flowering time

**Fig. 1:**
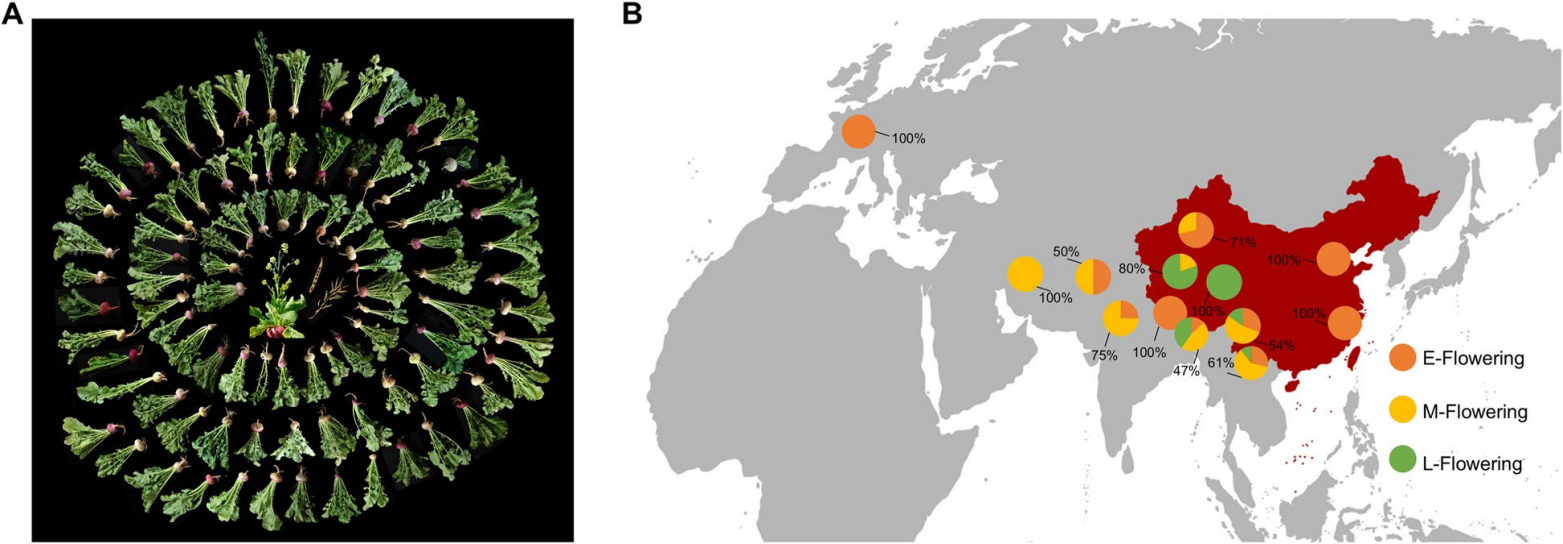
Analysis of flowering time among turnip accessions. **A**. Representative phenotypes of different turnip accessions, displaying the diversity of turnip accessions. **B**. The geographical distribution and flowering time of turnip accessions. The flowering time of the turnip accessions is categorized as early, middle, and late flowering. The accessions with flowering time less than 150 days are defined as early flowering (E-Flowering, orange color), 150–175 days as middle flowering (M-Flowering, yellow color), and more than 175 days as late flowering (L-Flowering, green color). The flowering types with the highest proportion in certain regions are labeled

**Fig. 2:**
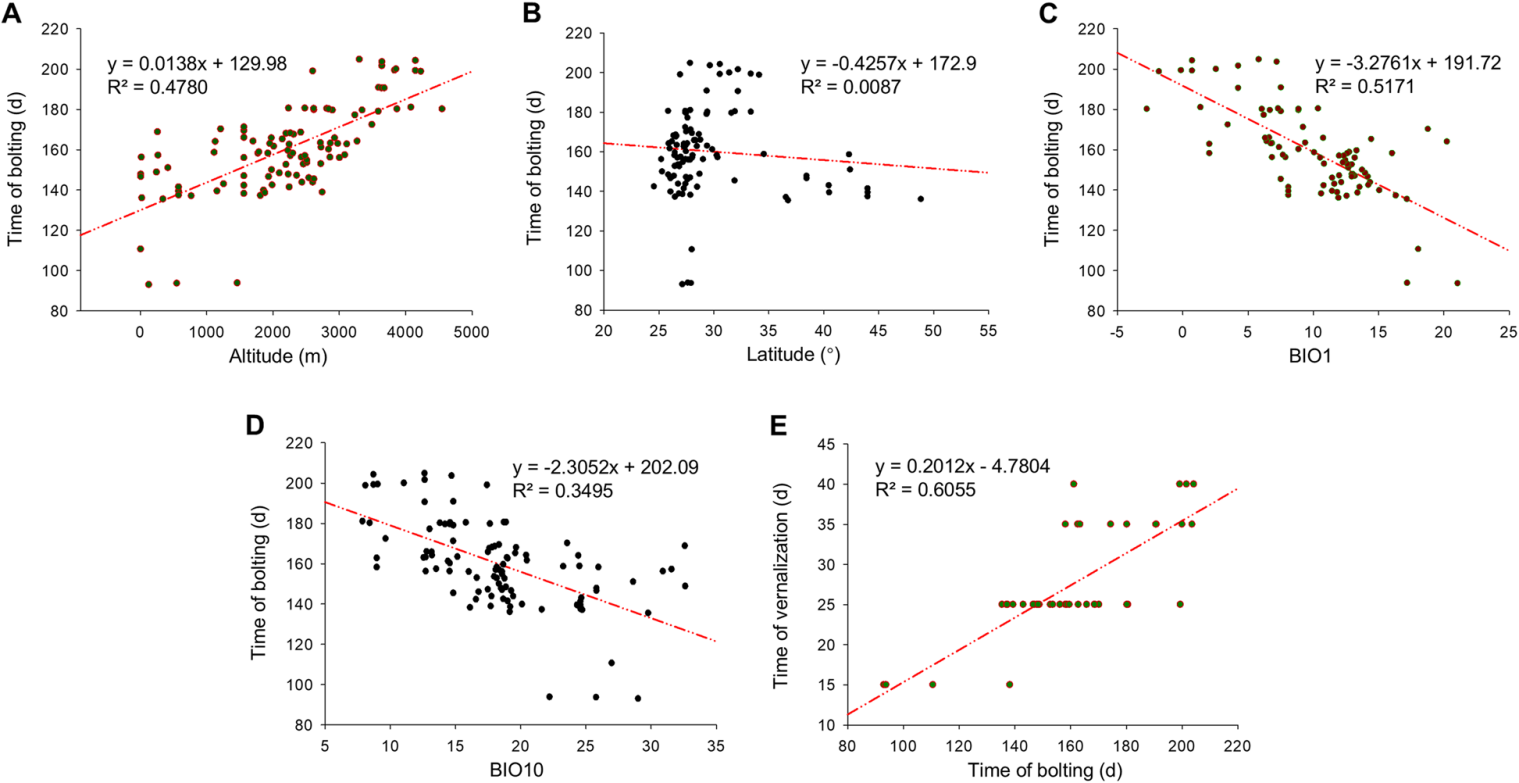
Correlation analysis of flowering time against altitude (**A**), latitude (**B**), ecological factors BIO1(annual mean temperature) (**C**), ecological factors BIO10 (mean temperature of warmest quarter) (**D**), and vernalization requirements (**E**) in turnip. “R” indicates the correlation index

In this study, we collected 104 turnip accessions from around the world (mainly from Europe, Central Asia, East Asia, and South Asia) and most of the local accessions cultivated in China to analyze their variations in flowering time (Fig. 1A). These accessions were grown in a greenhouse without cold exposure, and significant differences in flowering time were observed among turnips from different regions (Table S1). We mapped the geographic locations of these accessions and notated their flowering time at the corresponding locations to identify regional variations in flowering time. Our findings indicated obvious region-specific characteristics of flowering time: turnips from Central Asia and East Asia flowered mainly from days 150 to175 days (middle (M) flowering), those from the Qinghai-Xizang Plateau flowered much later (87% of accessions > 175 days, late (L) flowering), and turnips from low altitudes in China and Nepal flowered much earlier, from days 90 to 150 days (early (E) flowering) (Fig. 1B). Multiple regression analysis of flowering traits against two geographic variables (altitude and latitude) was conducted. The results revealed a close correlation between flowering time and the altitude of the turnip planting region (R^2^ = 0.4780) (Fig. 2A). The flowering time exhibited a strong altitudinal gradient: the higher the altitude, the later the flowering occurred. Specifically, the accessions collected from altitudes below 1000 m flowered much earlier than those from higher altitudes, whereas those from altitudes above 3000 m flowered much later. The relationship between latitude and flowering time did not show a simple correlation (Fig. 2B). Previous studies have suggested that the effect of the altitudinal gradient on flowering time can largely be explained by the decrease in temperature with increasing altitude (Suter et al. 2014; Méndez-Vigo et al. 2011). Analysis of the correlation between climate variables (19 ecological factors downloaded from the WorldClim database; www.WorldClim.org) and flowering time revealed that the “annual average temperature” (BIO1) (Fig. 2C, Fig. S1) and the “mean temperature of warmest quarter” (BIO10) (Fig. 2D) were strongly and negatively correlated with flowering time. In addition, vernalization requirements were observed among the turnip accessions, with greater demand for vernalization in high-altitude turnips with late flowering times (Fig. 2E). Therefore, the differences in flowering time among turnips appear to be strongly influenced by the climatic temperature along the altitudinal gradient.

### Role of *FRIa *and *FLC2* in influencing the variation of flowering time in turnip

In *Arabidopsis*, *FLC* is a central repressor of flowering, and FRI upregulates the expression of *FLC* to confer the winter-annual growth habit (Li et al. 2018). Previous studies have shown that variations in *FRI* and *FLC* are key determinants of differences in flowering time in *Arabidopsis*, and their homologs have been prime candidates affecting flowering time in other members of the Brassicaceae family (Schranz et al. 2002; Wang et al. 2006; Hepworth et al., 2020; Akter et al. 2021). In species of the *Brassica* genus, including turnips*, FRIa* and *FLC2* have been shown to be strong flowering repressors, (Xiao et al. 2013; Huang et al., 2018; Zheng et al., 2021; Zhang LP et al., 2024). Thus, to investigate potential variations in these two key genes, the genomic sequences of *FLC2* and *FRIa* were determined through whole-genome resequencing from turnip accessions. The results showed that *FLC2* was conserved among turnip accessions, with only one SNP in the 2.0-kb promoter and one nonsynonymous SNP in a few accessions (Table S2). Sanger sequencing of the 5.4-kb *FLC2* genomic sequence revealed that the sequence of *FLC2* was highly conserved among representative turnip accessions from different regions (Fig. S2). In contrast, a total of 18 variants (17 SNPs and 1 InDel), including 12 nonsynonymous variants, were identified at the *FRIa* locus among turnip accessions through whole-genome resequencing (Table S3). To further characterize the sequence variants in the *FRIa* gene, we cloned the 2.18-kb *FRIa* genomic sequence from the turnip accessions using PCR. Among the 104 materials, 90% of the accessions obtained the target sequences. Sanger sequencing identified a total of 10 nonsynonymous variants (snp1-10), 3 InDels and 1 Insert (Fig. S3 and Table S4), with 6 nonsynonymous variants overlapped with the resequencing data. We further detected the transcript levels of *FRIa* and *FLC2* in representative non-vernalized accessions. The results revealed a close correlation between the *FLC2* expression level and flowering time: the later the flowering, the higher the *FLC2* expression level observed (Fig. S4A). A positive correlation was also detected between the altitude of the collection site and the *FLC2* expression level (Fig. S4B). However, the *FRIa* expression level did not show significant correlation with flowering time (Fig. S5A) or with the altitude gradient (Fig. S5B). We also examined the correlation between the expression levels of the MADS-box transcription factors, such as *SHORT VEGETATIVE PHASE* (*SVP*) and *MADS AFFECTING FLOWERING* genes (*MAFs*), and the flowering time or altitude gradient, and we found that the expression levels of these genes were not closely correlated to the flowering time or altitude (Fig. S6, S7). These results demonstrate that *FLC2* expression levels influence flowering time in a dose-dependent manner in turnip, and as an upstream key regulator of *FLC2*, the *FRIa* nucleotide polymorphism that was observed in the turnip accessions may affect its function in regulating the *FLC2* expression and contribute to flowering time differences.

**Fig. 3:**
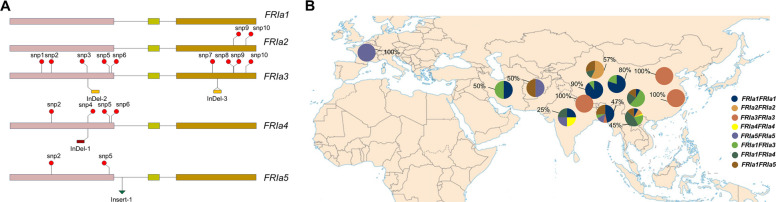
Sequence analysis of the *FRIa* alleles and genotypes among turnip accessions. **A**. Diagram of genomic structure of five *FRIa* alleles. Exons are shown as boxes and introns as black lines. The red circle indicates the SNP locations, the red and yellow small boxes indicate the InDels, and the green triangle indicates the Insert. **B**. Geographic distribution of eight *FRIa* genotypes. The highest percentages of each *FRIa* genotypes in certain region are labeled

We then analyzed sequence variations in the *FRIa* gene among the turnip accessions. Five distinct alleles (*FRIa1* ~ *5*) and eight genotypes were defined based on the sequence variants (Fig. 3A, Fig. S8). The results showed that none of the *FRIa* sequences experienced truncation, indicating that no nonfunctional *FRIa* alleles were present. In addition, the *FRIa* genotypes were associated with the cultivation locations of the turnip accessions, rather than being randomly distributed (Fig. 3B). Turnips carrying the *FRIa1FRIa1* genotype were predominantly observed in the Qinghai-Xizang region, representing 90.0% of the turnip genotypes in Xizang. Local accessions carrying the *FRIa3FRIa3* genotype were mainly found in Nepal and at low altitudes in China. The *FRIa2FRIa2* genotype was found mainly in Xinjiang of China. The *FRIa4FRIa4* and *FRIa5FRIa5* genotypes were only distributed in a few regions. Turnip accessions with *FRIa1FRIa3*, *FRIa1FRIa4* and *FRIa1FRIa5* genotypes were mainly from Yunnan and Sichuan of China, where the varied terrains and climates may contribute to high heterozygosity of the local accessions, as turnips are allogamous plants.

**Fig. 4:**
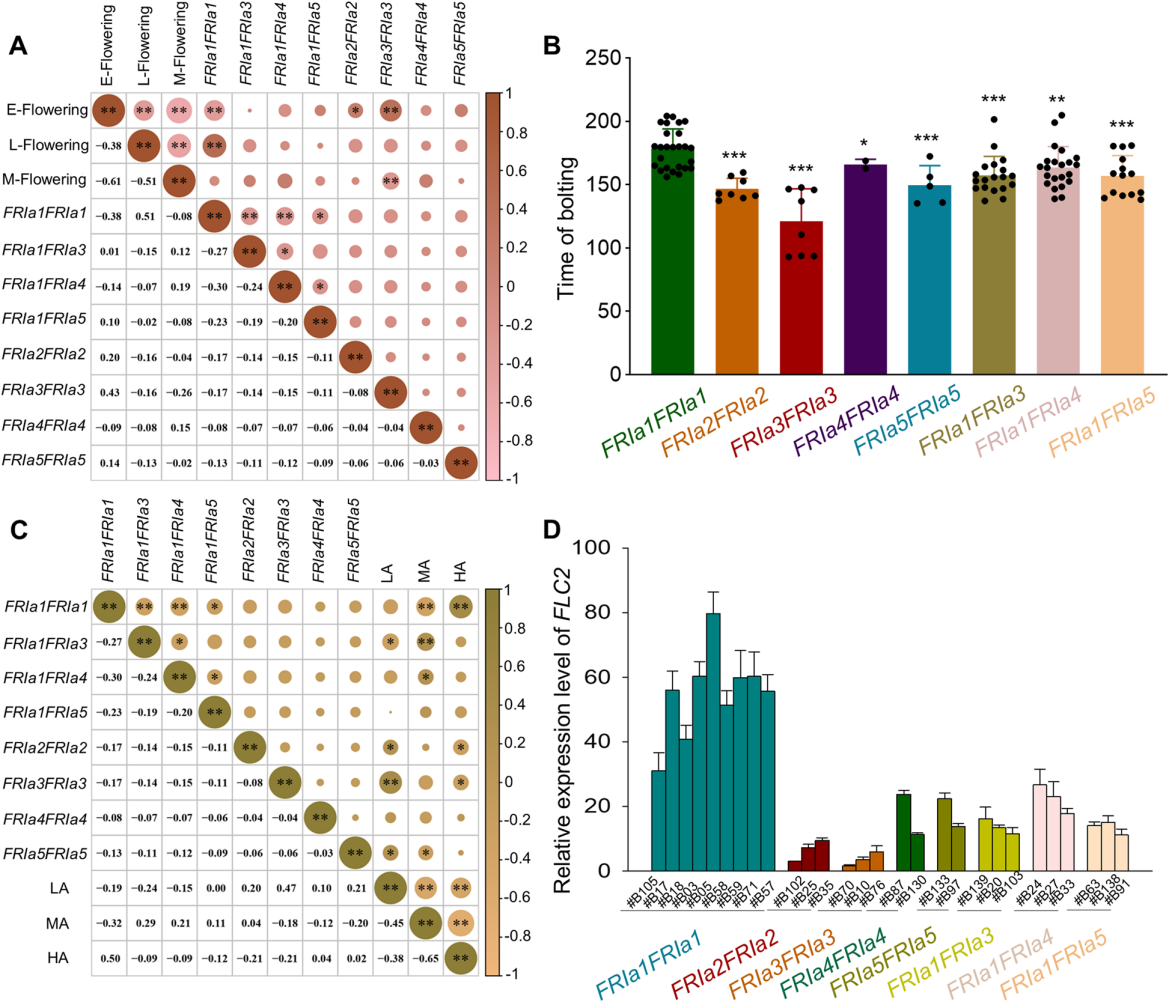
Association analysis between *FRIa* genotype and flowering time, altitude or *FLC2* expression levels. **A**. Correlation analysis of eight *FRIa* genotypes with flowering time (E-Flowering, M-Flowering, and L-Flowering) by Pearson factor (*r*). The upper right corner indicates the significance factor between them. *P < 0.05; **P < 0.01. The lower left corner indicates the Pearson coefficient (*r*) between them. For r, the closer to 1 or −1, the stronger the correlation (positive or negative). The right color gradient indicates the *r* value (−1 ~ 1).** B**. Flowering time of turnip accessions carrying each type of *FRIa* genotypes. Data are presented as mean ± *SD*; “*n*” indicates the number of turnip accessions carrying each genotype. *P < 0.05; **P < 0.01; ***P < 0.001, compared to *FRIa1FRIa1*. **C**. Correlation analysis of eight *FRIa* genotypes with altitudes (low altitude, LA; medium altitude, MA and high altitude, HA) by Pearson factor (*r*).** D**. Expression level of *FLC2* in representative turnip accessions carrying each type of *FRIa* genotypes. Twenty-day-old seedlings were harvested for RT‒qPCR assays. Data are presented as mean ± *SD*, *n* = 3

Correlation analysis between the *FRIa* genotype and flowering time revealed that *FRIa1FRIa1* was significantly related to late flowering time (*r* = 0.51), whereas *FRIa3FRIa3* was closely related to early flowering time (*r* = 0.43) (Fig. 4A, B). Moreover, *FRIa1FRIa1* was also strongly correlated with high altitude (*r* = 0.50) (Fig. 4C). Approximately 64.0% of the late-flowering phenotype could be explained by *FRIa1FRIa1*, indicating that *FRIa1FRIa1* might be associated with late flowering in local turnip accessions adapted to the Qinghai-Xizang Plateau. The function of FRI to repress flowering time is attributed to the induction of *FLC* transcription (Li et al. 2018). We thus further analyzed the influence of different *FRIa* genotypes on *FLC2* expression among turnip accessions. The representative turnips with the *FRIa1FRIa1* genotype presented the highest levels of *FLC2* expression, comparing with the other representative turnips, which was consistent with the late flowering observed (Fig. 4D). Analysis of *FRIa* transcription in non-vernalized turnips showed that there was no dominant correlation between *FRIa* expression levels and genotypes (Fig. S9).

Overall, we obtained comprehensive data on *FRIa* alleles and allelic genotypes, and conducted association analyses relating to geographical regions, flowering time, altitude, and *FLC2* expression levels. Notably, the *FRIa* alleles in the Qinghai-Xizang Plateau region had undergone variations compared with those at medium and low altitudes, possibly indicating the emergence of highly functional *FRIa* alleles.

### The* FRIa* allele in high altitude has a strong function for late flowering

**Fig. 5:**
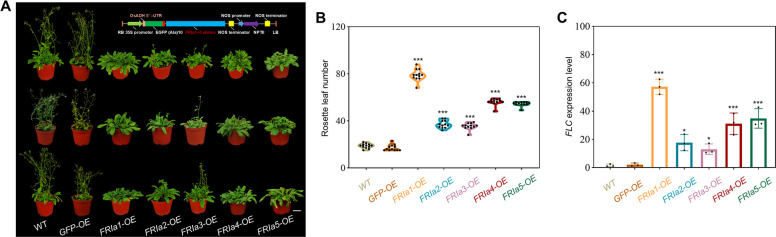
Functional validation of five *FRIa* alleles in regulation of flowering time. **A**. Representative phenotypes of wild-type (WT), transgenic *35S::GFP* plants (*GFP-OE*) and transgenic plants overexpressing *FRIa* alleles (*FRIa1* ~ *FRIa5-OE*) at 22 °C under long-day conditions. Scale bars = 1 cm. The physical maps of the regions between the RB and LB of vector construction are displayed above the transgenic plants. **B**. Flowering time of WT and transgenic plants. The flowering time was recorded as the number of rosette leaves. Data are presented as the mean ± *SD*, *n* = 12. ***P < 0.001. **C**. *FLC* expression levels in the leaves of WT and transgenic plants. Twelve-day-old seedlings were harvested for RT‒qPCR assays. Data are presented as the mean ± *SD*, *n* = 3. *P < 0.05; ***P < 0.001

To further validate the effects of the five *FRIa* alleles on flowering time, the full-length genomic sequence (2.1 kb) of each *FRIa* in turnips (Table S5) was ectopically overexpressed in *Arabidopsis thaliana* (ecotype Columbia, WT), which lacks natural *FRI* alleles. We did not carry out genetic validation of *FRIa* in turnips due to heterogamous pollination and the lack of natural *FRIa* mutant in turnips. The homozygous transgenic plants were obtained (Fig. S10) and planted under long-day conditions at 22 °C in soil to measure the flowering time and the number of rosette leaves at the opening of the first flower. Compared with the WT plants and the plants transformed with empty vector, all the transgenic lines presented significantly elevated levels of *FRIa* transcription and a late-flowering phenotype, although the flowering time varied significantly (Fig. 5A-B, Fig. S11A). Plants overexpressing *FRIa1*, which is distributed primarily in the Qinghai-Xizang Plateau region, had significantly more rosette leaves (79.00 ± 5.01) compared with those overexpressing any of the other four *FRIa* alleles, indicating more effective inhibition of flowering time. Plants overexpressing *FRIa2* (with 37.08 ± 3.26 rosette leaves) and *FRIa3* (with 35.42 ± 2.81 rosette leaves), which are distributed primarily in low-altitude areas, flowered later than the WT plants but much earlier than the plants overexpressing *FRIa1*. We also compared the *FLC* transcript levels among the five transgenic lines. While *FLC* expression levels were significantly higher in the transgenic lines than in the wild-type plants, there were differences among the transgenic lines, which was consistent with the differences in flowering time (Fig. 5C, Fig. S11B). Specifically, the transgenic *FRIa1-OE* plants exhibited the latest flowering time and the highest *FLC* transcription levels. These results indicated that the *FRIa* gene has evolved into a highly functional type *FRIa1* as turnip spreads to the Qinghai-Xizang Plateau, potentially enhancing domestication and adaptation to local environments.

### Key SNP variations in *FRIa1* confer strong functionality of *FRIa* and enhance late-flowering phenotype

**Fig. 6:**
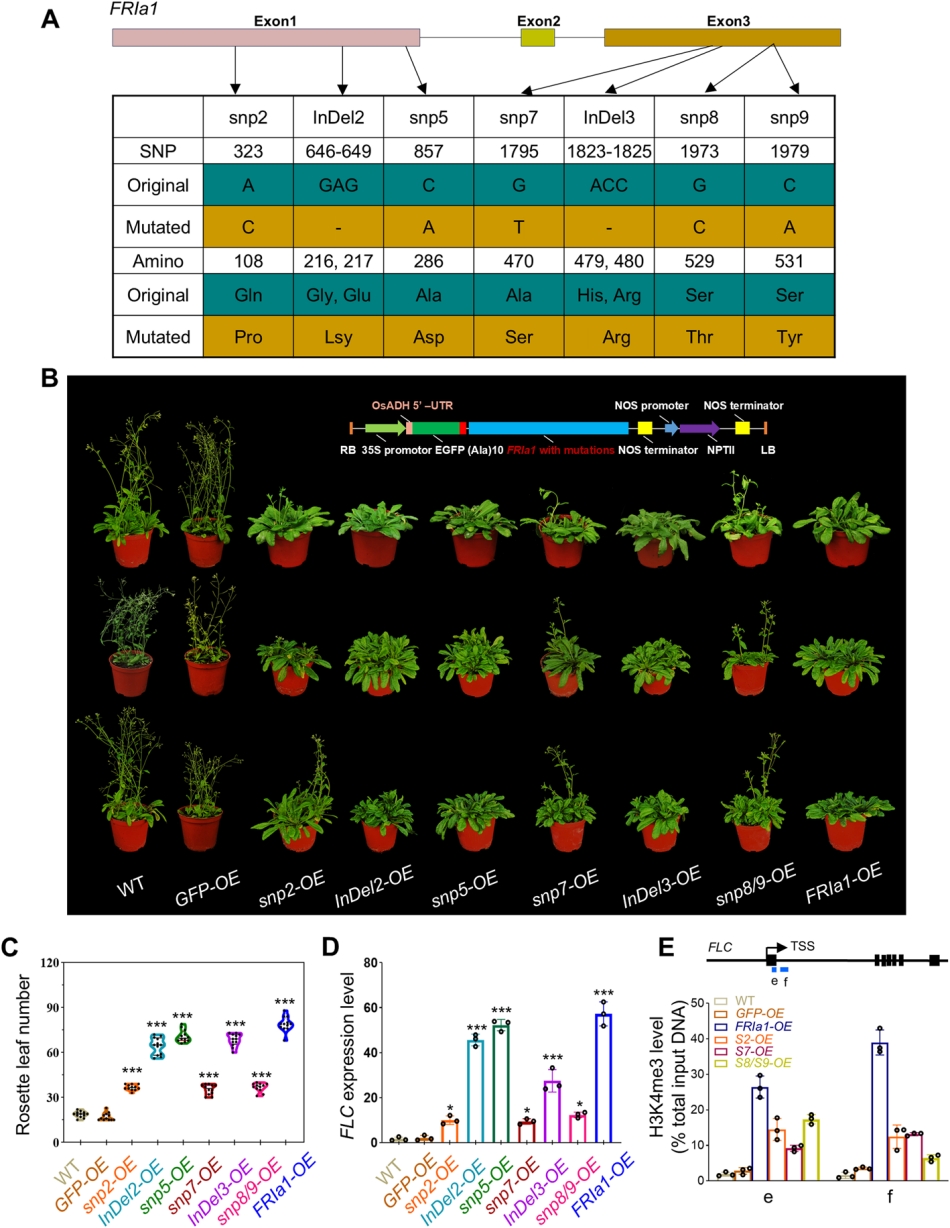
Functional identification of the key SNP variations in *FRIa*. **A**. The positions of SNPs and InDels in the *FRIa* gene region. Mutations in candidate nucleotides and corresponding amino acids are indicated. **B**. Representative phenotypes of WT, transgenic *35S::GFP* plants (*GFP-OE*) and transgenic plants overexpressing *FRIa1* (*FRIa1-OE*) or mutated *FRIa1-OE* (*snp2-OE*, *snp5-OE*, *snp7-OE*, *snp8/9-OE*, *InDel2-OE* and *InDel3-OE*) grown at 22 °C under long-day conditions. Scale bars = 1 cm. The physical maps of the regions between the RB and LB of vector construction are displayed above the transgenic plants. **C**. Flowering time of WT and transgenic plants. Data are presented as the mean ± *SD*, *n* = 12.*P <0.05; ***P < 0.001. **D**. *FLC* expression levels in the leaves of WT and transgenic plants. Twelve-day-old seedlings were harvested for RT‒qPCR assays. Data are presented as the mean ± *SD*, *n* = 3. ***P < 0.001. **E**. ChIP‒qPCR analysis of the H3K4me3 levels at the *FLC* locus in WT and transgenic plants. A Schematic diagram of the *FLC* genomic sequence. Exons are presented as black boxes, and the primer regions used for the ChIP-qPCR are indicated by blue boxes. Data are presented as the mean ± SD, *n* = 3

The functional variation in *FRIa* is caused mainly by locus mutations. Based on the association analysis between SNPs and flowering time of turnip (Fig. S12), five SNP variations and two InDels were validated, which may contribute to the functional differences in *FRIa* alleles, including snp2, snp5, snp7, snp8, snp9 and InDel-2 and InDel-3 (Fig. 6A). To determine whether these mutations are sufficient to account for the late flowering time observed in *FRIa1-OE*, we introduced the following mutations into *FRIa1*: an A-to-C substitution at snp2, a C-to-A substitution at snp5, a G-to-T substitution at snp7, a G/C-to-C/A substitution at snp8/9, deletion of the GAG nucleotides in InDel 2, and deletion of the ACC nucleotides in InDel3. The mutated sequences of *FRIa1* were then overexpressed in *Arabidopsis thaliana* (ecotype Columbia) (Fig. 6B, Fig. S13, 14). A previous study showed that some amino acid mutations of *FRI* can affect protein nuclear localization and stability (Zhang et al., 2020). In our study, GFP signals were detected in the root cell nuclei of transgenic plants overexpressing *FRIa1-GFP* and mutated *FRIa1-GFP* (Fig. S14). Furthermore, the subcellular localization of FRIa was unaffected by the mutations, with the protein detected in a punctate pattern within the nucleus, indicating that the mutations in *FRIa* did not affect protein stability or distribution. We further recorded the flowering time of the transgenic lines with mutated *FRIa1*. All the transgenic plants flowered later than the WT plants and the plants transformed with empty vector, whereas there were significant differences in flowering time among the transgenic plants (Fig. 6B, C, Fig. S15A). Specifically, plants expressing *FRIa1* with mutations at snp2, snp7 and snp8/9 flowered much earlier than the plants expressing the functional *FRIa1* (with 36.67 ± 1.87, 35.33 ± 3.37, and 36.75 ± 2.83 rosette leaves, respectively). The flowering time of *FRIa1-OE* transgenic plants with mutations at other loci was comparable to that of unmutated *FRIa1-OE* plants, implying that the snp2, snp7 and snp8/9 loci critically impact the functionality of *FRIa1*. Consistent with these findings, the *FLC* transcript levels were much lower in the transgenic plants overexpressing *FRIa1* with mutations at snp2, snp7 and snp8/9 than in the plants overexpressing functional *FRIa1*, but they were higher than the corresponding transcript levels in the wild-type *Arabidopsis* plants (Fig. 6D, Fig. S15B). Further analysis of the mutations of snp8, snp9 and snp10, respectively, indicated that both snp8 and snp9 showed an advance in flowering time (Fig. S16), but simultaneous snp8/9 mutations were more effective in promoting flowering time compared with single point mutations (Fig. 6C). These experiments confirmed that the snp2, snp7 and snp8/9 loci in the *FRIa1* play critical roles in influencing FRIa function and activation of *FLC* transcription, resulting in delayed flowering.

FRI-mediated histone H3K4 trimethylation (H3K4me3), which binds in the chromatin region surrounding the transcription start site (TSS) of *FLC*, is crucial for activating *FLC* expression to delay flowering (Zhang et al., 2020). We further performed ChIP‒qPCR analysis to investigate whether mutations in *FRIa1* influence histone modification in the *FLC* chromatin. We detected H3K4me3 histone modification in two regions of *FLC*, which have been shown to be closely associated with high-level *FLC* expression (Li et al. 2018). Compared with that in WT *Arabidopsis*, a considerable increase in H3K4me3 around the *FLC* TSS region was observed in transgenic plants overexpressing *FRIa1* (Fig. 6E). However, in transgenic plants overexpressing *FRIa1* with mutations at snp2, snp7 and snp8/9, the levels of H3K4me3 in *FLC* were significantly decreased. These results suggest that the variation at the *FRIa*-snp2, snp7 and snp8/9 loci affects the histone modification levels of the *FLC* gene. We further analyzed the correlation between these SNP variations in turnip accessions and altitudes. The results showed that the snp2, snp7 and snp8/9 loci were closely related to altitudes (Fig. S17).

Furthermore, we analyzed the potential structural changes of the FRIa protein caused by variations at the four key SNP sites at the amino acid level (Fig. S18). After obtaining a predicted protein structure with high structural score of FRIa1, the four SNPs were introduced into the protein (Fig. S18A). Analyses of the hydrophobicity, atomic charge, intermolecular interactions, and secondary structure of amino acids revealed that the mutations at the four key sites caused alterations at the amino acid level of the predicted FRIa1 protein. Specifically, mutations at positions snp2 (G108P) and snp7 (A470S) caused obvious changes in the hydrophobicity (Fig. S18B) and atomic charge (Fig. S18C), and the electrostatic interaction between molecules increased after the snp9 (S531T) mutation was introduced (Fig. S18D). Secondary structure analysis suggested that the mutation of glutamine to proline at snp2 might lead to the change of β-turn between proline at snp2 and prolines at position 105, 106, and 107. The mutation at position snp7 caused a subtle change in the structure of the α-helix in this segment, making the structure of the α-helix tighter. The mutations at positions snp8 and snp9 did not show significant impacts on the secondary structure, but the mutations might change the stability of the protein structure by altering the intermolecular interactions within the protein (Fig. S18E). These findings suggest that these alterations at the amino acid level may affect the interactions between FRIa and other partner proteins, ultimately impacting the function of FRIa. Together, these results indicate that the amino acids 108 Gln (snp2 CAA), 470 Ala (snp7 GCA), 529 Ser (snp8 AGT), and 531 Ser (snp9 TCT) in the *FRIa1* protein of local turnip accessions growing in the Qinghai-Xizang region play a significant role in delaying flowering for adaptation to high altitudes.

To investigate whether these four SNP sites are conserved and crucial for flowering time variations in the Brassicaceae family, we further examined the conservation of these four SNP variations in different species of the Brassicaceae family through aligning *FRI* homologues with the *FRIa1* sequence (Fig. S19). We obtained the functional *FRI* sequence in *Arabidopsis thaliana*, *Arabidopsis lyrate*, *Thlaspi arvense*, *Capsella* *rubella*, *Brassica oleracea* (genome B), *Brassica juncea* (genome AB), *Brassica napus A* (genome AC), *Brassica carinata* (genome BC) and ecotypes or accessions in these species, based on the previous studies (Kuittinen et al. 2008; Wang et al., 2011; Irwin et al. 2012; Fadina et al. 2013; Fadina and Khavkin 2014; Yang et al., 2018; Zhang et al., 2020; Geng et al. 2021). We found that the four key SNPs in turnip were not responsible for flowering variations in other species. In *Arabidopsis thaliana* and *Arabidopsis lyrate*, snp2, snp7 and snp9 were not conserved as in *FRIa1*, but snp8 was conserved. However, we noticed that these four nucleotide sites were not the SNP variation sites account for the differences in flowering among *A. thaliana* and *A. lyrate* accessions, and snp8 was conserved in both of the late- and early- flowering ecotypes. In *B. napus* and *B. oleracea*, the sequence of *FRIa* has been reported to play a role in flowering-time variation of winter, semi-winter and spring accessions. However, the four SNPs of turnip did not associate with the flowering time variations of *B. napus* and *B. oleracea* accessions. It was found that SNPs198, −294, −348, −417, and −1790 were associated with flowering time variation in 95 accessions in *B. napus* (Wang et al., 2011). This can be understood as turnip, *B. napus* or *Arabidopsis* experienced different growth environments and migration patterns, which may have resulted in different evolutionary dynamics.

## Discussion

**Fig. 7:**
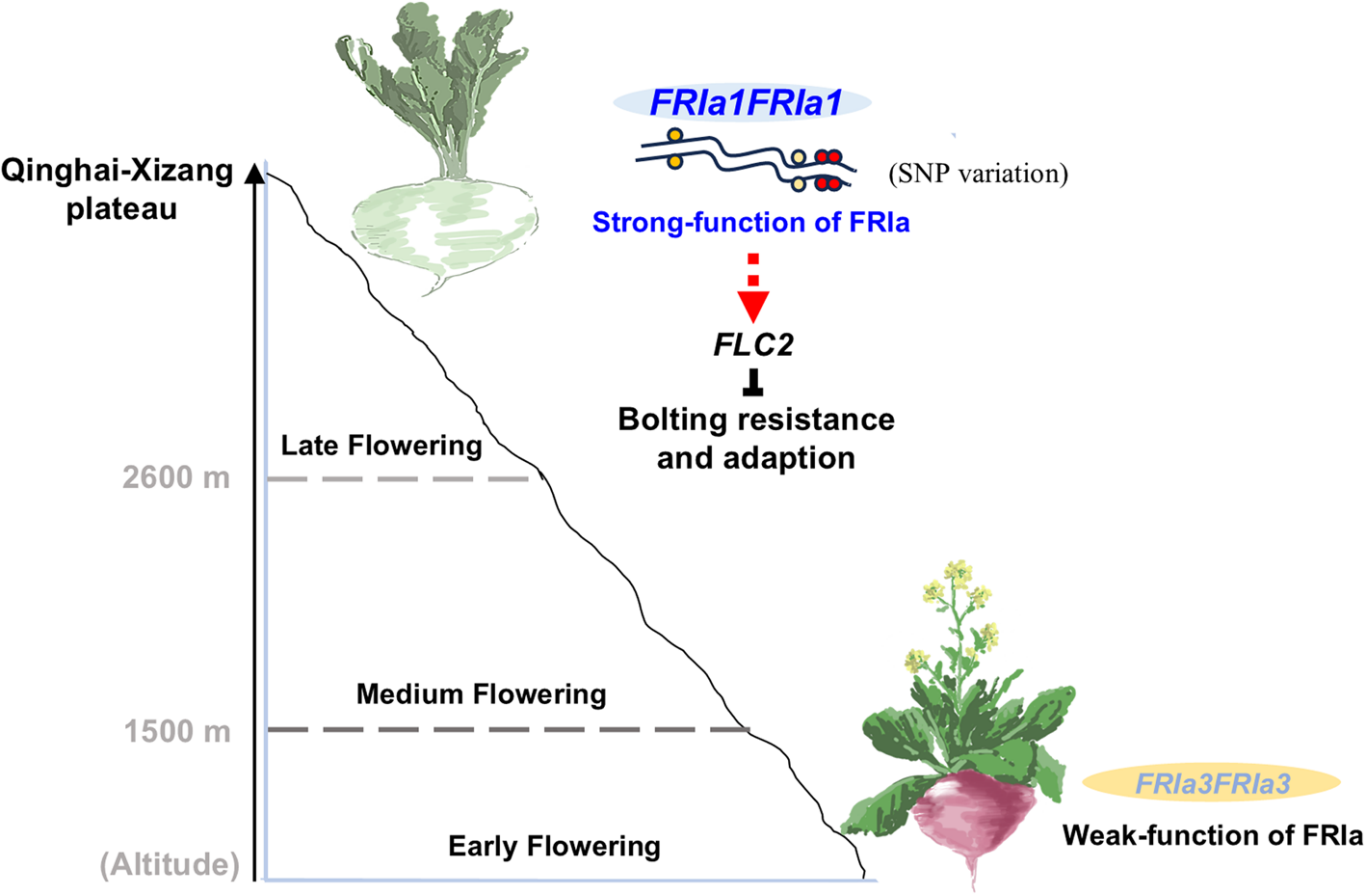
A model of *FRIa* variation in regulating flowering time in turnip. The flowering time of turnips exhibits adaptive traits across altitude gradients. High-altitude *FRIa* alleles have strong function for late flowering and are thought to contribute to turnip adaptation to the Qinghai-Xizang Plateau. Four specific SNP variations in *FRIa* were determined to be critical for its strong function in delaying flowering time

Flowering time is a key trait influencing plant dispersal across various altitudes. Moreover, flowering time is also impacted by the adaptation of plants to regional climatic conditions. As a crucial *Brassica* L. species, changes in the flowering time of turnip significantly influence its dispersal and adaptation to local environmental conditions. Studies have shown that plants at high altitudes undergo adaptive genetic evolution to survive in harsh environmental conditions. Flowering time is a critical developmental stage in seasonal alpine environments: premature flowering increases the risk of exposure to low temperatures and subsequent mortality. A few plants can survive in high-altitude plateau regions. Studies have found that plants have evolved diverse strategies for adapting to the high-altitude conditions. For example, *Thlaspi arvense*, which grows on the Xizang Plateau, has a short growing season and flowers relatively early. Our findings clearly indicate a positive correlation between the flowering time of turnip accessions and altitude, and turnip accessions on the Qinghai-Xizang Plateau flowered late as an adaptation to the high altitude. In this study, we focused on the variation in flowering time among turnip accessions and the role of the key gene *FRIa* in regulating floral transitions. We propose that the *FRIa* alleles in the accessions from Qinghai and Xizang are advantageous for the adaptation to the high-altitude climate, representing adaptive variations driven by strong selection that induces changes in flowering time (Fig. 7). This analysis provides an important example of the highly functional variation in *FRIa* driving adaptive flowering time under natural selection and domestication conditions with increasing altitude.

Understanding the molecular basis of flowering time variation provides a possibility for the direct selection of advantageous allele genes (Wang et al., 2011). Previous studies in natural *Arabidopsis* populations have focused primarily on the variation in flowering time between functional and nonfunctional alleles of *FRI* with changes in latitude (Stinchcombe et al. 2004). In this study, the flowering time of turnip was significantly related to the altitude gradient but was independent of the latitude gradient. This discrepancy may be attributed to differences in the selective driving forces between domesticated crops and natural populations, as well as to environmental differences in plant breeding. For example, high-latitude *Arabidopsis* populations evolved rapid flowering cycles as they expanded toward lower latitudes, resulting in multiple evolutionary variations in *FRI* alleles into nonfunctional alleles (Johanson et al. 2000; Gazzani et al. 2003; Stinchcombe et al. 2004). However, turnip, as a winter annual crop, evolved a delayed flowering habit for adaptation to extremely high altitudes during its spreading to the Qinghai-Xizang Plateau, leading to evolutionary variations in *FRIa* alleles toward highly functional types. In *T. arvense*, the *FLC* gene is subjected to strong natural selection in high-altitude environments, resulting in the loss of *FLC* function due to single-base mutations and subsequent adapting to the plateau environment via early flowering (Geng et al. 2021). Thus, plants have evolved different flowering strategies to adapt to high-altitude environments based on unique variations in key flowering genes. Though turnips are outcrossed plants, *FRIa* genotypes in turnips from Xizang at altitudes of more than 3500 m exhibit high homozygosity and carry highly functional *FRIa* alleles. Therefore, we believe that *FRIa* is associated to a unique adaptive pathway during the evolution of turnip populations as they spread to the Qinghai-Xizang Plateau. Interestingly, weakly functional *FRIa* alleles in turnip accessions from Nepal and southern China may have undergone parallel evolution, as turnips from these two regions carry the same homozygous *FRIa* alleles and similar flowering traits, despite substantial morphological differences between turnip materials from these two regions. These independent highly functional and weakly functional *FRIa* alleles exhibit stable homozygous genotype variations in outcrossed turnips, implying that these alleles are maintained throughout the differentiation of turnip accessions. Selection pressure on *FRIa* may occur during turnip dispersal along altitudinal gradients, selecting for advantageous functional *FRIa* alleles in high-altitude areas, where *FRIa* alleles significantly delay flowering time to a greater extent. It is evident that turnip *FRIa* variation may enhance reproductive adaptability in specific geographical environments by preserving different degrees of functionality, thereby conferring selective advantages and aiding in completion of the life cycle in cultivation areas.

How the key SNP sites in the *FRIa* sequence influence turnip flowering time remains unclear. In this study, four SNPs (snp2, 7, and snp8/9) in *FRIa* were found to be functionally associated. Amino acid substitutions generated differences in FRIa function that were significantly correlated with the delayed flowering. A previous study has indicated that two mutations determine the stability and cellular localization of the *FRI* protein in *Arabidopsis* (Zhang et al., 2020). We found that mutations in *FRIa* did not alter the stability or localization of the FRIa protein, but these variant sites affected the *FLC* expression levels and H3K4me3 modifications in the *FLC* chromatin. Previous studies have found that both the activation and inhibition of *FLC* are regulated by chromatin epigenetic modifications (Hepworth and Dean 2015). In winter-annual *Arabidopsis*, FRI-mediated activation of *FLC* involves H3K4me3 at *FLC* chromatin, which is essential for maintaining high *FLC* expression levels and late-flowering phenotypes. During vernalization, downregulation of *FLC* expression is associated with a directional chromatin state transition from the active mark H3K4me3 to the repressive mark H3K27me3, and subsequent suppression of *FLC* activates *FT* expression, ultimately initiating flowering (Berry and Dean 2015). Similarly, in *B. rapa*, the *FLC* genes exhibit H3K4me3 accumulation on their chromatin under non-vernalized conditions, while H3K27me3 deposition is increased after vernalization (Kawanabe et al. 2016; Akter et al. 2019). Differences between SNP variants of *FRIa1* and *FRIa3* in the corresponding gene regions may account for the functional heterogeneity of FRIa in regulating *FLC* expression and chromatin modifications, which underlies the variation in flowering time among turnip accessions. These observations suggest that long-term domestication and selection may drive variation in the *FRIa* gene and its functionality. Studies in *Arabidopsis* suggest that different regions of the FRI protein variably affect protein function and participate in interactions with different proteins within the FRI complex (Risk et al. 2010; Choi et al. 2011). Thus, amino acid variations in turnip FRIa protein may affect its ability to interact with potential complex component proteins, and the observed decrease in *FLC* expression levels and H3K4me3 enrichment may result from this effect. These key sites in *FRIa1* alleles can serve as candidate sites for functional analysis, aiding turnip breeding for resistance to early bolting through site-directed mutagenesis and the development of functional markers for turnip flowering time. In summary, our findings demonstrate that variations in *FRIa* play a significant role in influencing the flowering time of turnip, which holds potential for further domestication of Brassicaceae plants as well as for molecular breeding aimed at improving agronomic traits.

## Materials and methods

### Plant materials and growth conditions

The 104 turnip accessions used in this study were collected from the main turnip cultivation areas representing different geographical regions and altitudes across the world. These materials comprised 27 accessions originating from countries including France, Bhutan, Nepal, Iran, India, and Afghanistan, as well as 77 accessions from cultivation regions in China. The turnip seeds were sown in petri dishes that contained two pieces of filter paper and incubated in the dark at 22 ± 1 °C for 3 days until germination. The seedlings were subsequently transferred to warm and long-day (LD) conditions (16 h light and 8 h dark) at 23 °C. The corresponding transgenic *Arabidopsis* lines overexpressing different *FRIa* alleles and mutagenized *FRIa* genes were obtained, and seeds were sown on 1/2-strength Murashige and Skoog (MS) medium plates supplemented with kanamycin (30 mg/L). The transgenic seedlings were subsequently transferred to a greenhouse under similar planting conditions as those for turnip.

### Measurement of flowering time

The turnip flowering time was recorded as the number of days from sowing to blotting. At least five independent plants were used to analyze the flowering time for each accession. To clarify the flowering time, the accessions with flowering time less than 150 days were defined as early flowering, 150–175 days as middle flowering, and more than 175 days as late flowering (Wang et al., 2011; Ahn et al., 2024). Flowering time of the *Arabidopsis* plants was recorded as the number of days from sowing to the appearance of the first flower, and the total number of rosette leaves was also recorded. At least twelve independent plants were used to analyze the flowering time of each transgenic line.

### Resequencing and detection of variations

DNA was extracted from 104 accessions and used for the preparation and sequencing of Illumina libraries (350 bp fragments) on the Illumina HiSeq platform. Reads were discarded if they contained ≥ 10% unidentified nucleotides, > 10 nucleotides aligned to the adapter, or > 50% bases with a Phred quality score < 5. Filtered data were mapped to the turnip reference genome using BWA (v0.7.8) (Li et al., 2009a) with the following parameters: mem –t 4 –k 32 –M. Duplicate reads were deleted using SAMtools (v0.1.19) (Li et al., 2009b). Variations were identified using the Genome Analysis Toolkit (McKenna et al. 2010), and high-quality SNPs and insertions/deletions (INDELs) were identified using the HaplotypeCaller module. Combining the GVCF files generated for each sample with the following parameters yielded the final SNP and INDEL files: individual depth ≥ 4, individual genotype quality ≥ 5, minor allele frequency (MAF) ≥ 0.05, deletion rate ≤ 0.1, and heterozygosity < 0.1.

### Analysis of FRIa alleles and genotypes

The *FRIa* and *FLC2* alleles and SNP positions were analyzed. The chromosomal positions of the variant loci were determined using SNP files. In addition, Beagle was used for phasing genotypes to elucidate the distribution of allele codes among loci to generate haplotype files, which were subsequently visualized. Meanwhile, variations in the *FRIa* and *FLC2* sequence among turnip samples were analyzed through Sanger sequencing. Young leaves (*n* = 5) were collected from each turnip plant grown in a greenhouse. The genomic DNA isolated from each sample using the CTAB method served as the template for PCR amplification of the 2.1 kb *FRIa* genomic sequence and the 5.4 kb *FLC2* genomic sequence using gene-specific primers. The primers used are listed in Table S6.

### Gene cloning, construct generation and plant transformation

To produce the *FRIa-GFP* constructs, the full-length *FRIa* genomic sequences were amplified by PCR using specific primers. After the amplified *FRIa* fragments were sequenced, they were inserted into the pRI101-GFP vector (Clontech, PA, USA) that was digested with SalI and EcoRI to express GFP-FRIa fusion proteins under the control of the cauliflower mosaic virus 35S promoter. To mutagenize *FRIa*, the snp2 (snp323C), snp5 (snp857A), snp7 (snp1795T), snp8/9 (snp1973C/1979A), snp8 (snp1973C), snp9 (1979A), snp10 (2083C), InDel2 (InDel646-649) and InDel3 (InDel1823-1825) mutations were introduced into the *FRIa1-GFP* construct using the Mut Express® MultiS Fast Mutagenesis Kit (Vazyme, Nanjing, China). The *FRIa-GFP*, mutagenized constructs and pRI101-GFP empty vector were inserted into *Agrobacterium tumefaciens* EHA105 cells via electroporation for the subsequent transformation of wild-type *Arabidopsis* Columbia (Col-0). The primers used are listed in Table S6.

### Subcellular localization analysis

The transgenic seedlings carrying *FRIa-GFP* and mutagenized *FRIa-GFP* were grown in plates positioned vertically and incubated under LD conditions for 10 days. Seedling roots were immersed in a 10 mg/ml propidium iodide (PI) solution for 45 s and then rinsed in water. The root tips were examined using an Olympus FluoView confocal microscope (FV1000; OLYMPUS, Tokyo, Japan). The GFP fluorescence of the fusion protein was detected at a wavelength of 488 nm, and the PI signal was detected at a wavelength of 546 nm.

### RT‒qPCR

Total RNA was extracted using the Eastep® Super Total RNA Extraction Kit (Promega, WI, USA). First-strand cDNA was synthesized from 2.0 mg of the DNase-treated RNA using the 5 × All-In-One RT MasterMix (with AccuRT Genomic DNA Removal Kit) (ABM, Wuhan, China). The RT-qPCR analysis was performed using the EvaGreen qPCR MasterMix (ABM, Wuhan, China) and the StepOnePlus™ Real-Time PCR System (Applied Biosystems, MA, USA). The *Arabidopsis ACTIN2* (F: 5'-GCTGAGAGATTCAGATGCCCA-3'; R: 5'-GTGGATTCCAGCAGCTTCCAT-3') and turnip *TUB2* (F: 5'-AGGCGTGTGAGTGAGCAGTT-3'; R: 5'-CATCTCGTCCATTCCTTCACCTGT-3') genes were used as internal controls to normalize the expression levels of the target genes according to the 2^−ΔΔCt^ method. Each sample was completed using at least three technical replicates and three biological replicates. The primers used are listed in Table S6.

### ChIP‒qPCR analysis

The ChIP assay was performed using the SimpleChIP Kit (Cell signaling, MA, USA). Briefly, 0.2 g samples of 2-week-old *Arabidopsis* seedlings were collected and treated with 1% formaldehyde buffer to promote cross-linking, after which the nuclei were extracted and sonicated. Immunoprecipitation was performed using anti-trimethyl H3K4 antibodies (Sigma‐Aldrich, MO, USA). The DNA was extracted and precipitated using standard procedures. Both immunoprecipitated DNA and input DNA were recovered using water and analyzed by RT**‒**qPCR as described above. The ChIP efficiency was normalized according to the corresponding DNA input values (percent input = 2% × 2(C[T] 2% input sample − C[T] IP sample). The primers used are listed in Table S6.

### Statistical analysis

Statistical analysis was performed using one-way ANOVA and Student’s *t*-test (*P < 0.05, **P < 0.01, and ***P < 0.001). Data analysis was conducted using Microsoft Excel 2019 and GraphPad Prism 8.0. The correlation analysis was conducted using R package by Spearman and Kendall and plotting with corrplot.

## Supplementary Information

Additional file 1: Fig. S1 Correlation analysis of flowering time against ecological factors BIO2-9, BIO11-19. Fig. S2 Multiple alignments of 5.4-kb *FLC2* genomic sequence among different turnip accessions. Fig. S3 Gene structure of *FRIa* and nucleotide mutated sites detected among turnip accessions. Fig. S4 Correlation analysis between *FLC2* expression levels with flowering time (A) or altitude (B) among most of turnip accessions. Fig. S5 Correlation relationships between *FRIa* expression levels with flowering time (A) or altitude (B) among most of turnip accessions. Fig. S6 Correlation relationships between the expression levels of MADS-box genes and flowering time among different turnip accessions. Fig. S7 Correlation relationships between the expression levels of MADS-box genes and altitude among different turnip accessions. Fig. S8 Sequence analysis of the *FRIa* genotypes among turnip accessions. Fig. S9 Expression levels of *FRIa* in representative turnip accessions carrying each type of *FRIa* genotypes. Fig. S10 Identification of transgenic plants overexpressing the *FRIa* alleles. Fig. S11 Functional validation of five *FRIa* alleles in regulating flowering time. Fig. S12 Association analysis between SNPs and flowering time of turnip accessions carrying different SNP loci of *FRIa* alleles. Fig. S13 Expression levels of *FRIa* in WT and transgenic plants overexpressing *FRIa1* (*FRIa1-OE*) or mutated *FRIa1-OE*. Fig. S14 Subcellular localization of GFP-FRIa fusion proteins in root tips of transgenic seedlings overexpressing *FRIa1-OE* or mutated *FRIa1-OE*. Fig. S15 Functional identification of the key SNP variations in *FRIa*. Fig. S16 Functional validation of snp8, snp9 and snp10 variations in *FRIa*. Fig. S17 Association analysis between SNPs of the *FRIa* alleles and altitudes of the turnip planting regions. Fig. S18 The potential structural changes of FRIa protein caused by the key four SNP sites at the amino acid level. Fig. S19 Multiple alignments of *FRIa* gene sequences with *FRI* homologues in the Brassicaceae family.

Additional file 2: Table S1. Information of the *Brassica rapa* ssp. *rapa* samples used in this study. Table S2. *FLC2* SNP variants detected by resequencing data. Table S3. *FRIa* SNP variants detected by resequencing data. Table S4. *FRIa* nonsynonymous variants detected by Sanger sequencing. Table S5. Sequence of five *FRIa* alleles. Table S6. List of primers used in this study.

## Data Availability

The data will be available from the corresponding author upon reasonable request. Raw Illumina data of resequencing have been deposited in the National Tibetan Plateau Third Pole Environment Data Center under accession number XDA2004010306 (https://data.tpdc.ac.cn/zh-hans/data/70d73bf9-6d82-4437-8d66-5cf87584f89f).

## Abbreviations

FRIa: FRIGIDA a
FLC: FLOWERING LOCUS C
SNP: Single Nucleotide Polymorphism
InDel: Insertion-Deletion
H3K4me3: Tri-methylation of lysine 4 on histone H3
RT**‒**qPCR: Quantitative real-time polymerase chain reaction
ChIP**‒**qPCR: ChIP**‒**Quantitative Polymerase Chain Reaction
TSS: Transcription Start Site
Gln: Glutamine
Ala: Alanine
Ser: Serine
LD: Long Day
MS: Murashige and Skoog
DNA: Deoxyribonucleic Acid
CTAB: Cetyltrimethylammonium Bromide
PI: Propidium Iodide
GFP: Green Fluorescent Protein
ChIP: Chromatin Immunoprecipitation Assay
ANOVA: Analysis of Variance
