# Supplementary material for: A functional FRIa variant drives turnip adaptation to the Qinghai-Xizang Plateau
Source: Mol Hortic. 2026 Jan 12;6:6. doi: 10.1186/s43897-025-00185-9 (PMC12794460; doi:10.1186/s43897-025-00185-9)
Supplement: Supplementary file 1 — Additional file 1: Fig. S1 Correlation analysis of flowering time against ecological factors BIO2-9, BIO11-19. Fig. S2 Multiple alignments of 5.4-kb FLC2 genomic sequence among different turnip accessions. Fig. S3 Gene structure of FRIa and nucleotide mutated sites detected among turnip accessions. Fig. S4 Correlation analysis between FLC2 expression levels with flowering time (A) or altitude (B) among most of turnip accessions. Fig. S5 Correlation relationships between FRIa expression levels with flowering time (A) or altitude (B) among most of turnip accessions. Fig. S6 Correlation relationships between the expression levels of MADS-box genes and flowering time among different turnip accessions. Fig. S7 Correlation relationships between the expression levels of MADS-box genes and altitude among different turnip accessions. Fig. S8 Sequence analysis of the FRIa genotypes among turnip accessions. Fig. S9 Expression levels of FRIa in representative turnip accessions carrying each type of FRIa genotypes. Fig. S10 Identification of transgenic plants overexpressing the FRIa alleles. Fig. S11 Functional validation of five FRIa alleles in regulating flowering time. Fig. S12 Association analysis between SNPs and flowering time of turnip accessions carrying different SNP loci of FRIa alleles. Fig. S13 Expression levels of FRIa in WT and transgenic plants overexpressing FRIa1 (FRIa1-OE) or mutated FRIa1-OE. Fig. S14 Subcellular localization of GFP-FRIa fusion proteins in root tips of transgenic seedlings overexpressing FRIa1-OE or mutated FRIa1-OE. Fig. S15 Functional identification of the key SNP variations in FRIa. Fig. S16 Functional validation of snp8, snp9 and snp10 variations in FRIa. Fig. S17 Association analysis between SNPs of the FRIa alleles and altitudes of the turnip planting regions. Fig. S18 The potential structural changes of FRIa protein caused by the key four SNP sites at the amino acid level. Fig. S19 Multiple alignments of FRIa gene sequences with FRI hom [file 43897_2025_185_MOESM1_ESM.docx]

**A functional *FRIa* variant drives turnip adaptation to the Qinghai-Xizang Plateau**

Yan Zheng^1,2,3a^, Danni Yang ^1,2,3a^, Landi Luo ^1,2^, Xin Yin^1,2,3^, Xingyu Yang^1,2,3^, Yunqiang Yang^1,2,3*^, Xiangxiang Kong^1,2,3*^, Yongping Yang^1,2,3*^

^1^ CAS Key Laboratory of Tropical Plant Resources and Sustainable Use, Xishuangbanna Tropical Botanical Garden, Chinese Academy of Sciences, Menglun, Mengla, 666303, Yunnan, China

^2^ Germplasm Bank of Wild Species, Yunnan Key Laboratory for Crop Wild Relatives Omics, Kunming Institute of Botany, Chinese Academy of Sciences, Kunming, Yunnan 650201, China

^3^ University of Chinese Academy of Sciences, Beijing 100049, China

^a^ These authors contributed equally to this work.

^*^ Corresponding authors: Yongping Yang (yangyp@xtbg.ac.cn), Xiangxiang Kong (kongxiangxiang@xtbg.ac.cn) and Yunqiang Yang (yangyunqiang@xtbg.ac.cn).

**Core**

*FRIa* variation plays a key role in the adaptive trait of flowering time in turnip. High-altitude *FRIa* alleles in turnip are described as highly functional alleles that contribute to late flowering of turnip during adaptation to the Qinghai-Xizang Plateau. Four specific SNP variations in *FRIa* in turnip were identified to be critical for its function in delaying flowering time.

**Supplementary Figures**

**Fig. S1** Correlation analysis of flowering time against ecological factors BIO2-9, BIO11-19. BIO2, mean diurnal range; BIO3, isothermality; BIO4, temperature seasonality; BIO5, max temperature of warmest month; BIO6, min temperature of coldest month; BIO7, temperature annual range; BIO8, mean temperature of wettest quarter; BIO9, mean temperature of driest quarter; BIO11, mean temperature of coldest quarter; BIO12, annual precipitation; BIO13, precipitation of wettest month; BIO14, precipitation of driest month; BIO15, CV of precipitation; BIO16, precipitation of wettest quarter; BIO17, precipitation of direst quarter; BIO18, precipitation of warmest quarter; BIO19, precipitation of coldest quarter; “R” indicates the correlation index.

**Fig. S2** Multiple alignments of 5.4-kb *FLC2* genomic sequence among different turnip accessions. The ATG starting site is marked.

**Fig. S3** Gene structure of *FRIa* and nucleotide mutated sites detected among turnip accessions. Exons are shown as boxes and introns as black lines. The red circle indicates the SNP locations, the red and yellow small boxes indicate the InDels, and the green triangle indicates the Insert.

**Fig. S4** Correlation analysis between *FLC2* expression levels with flowering time (**A**) or altitude (**B**) among most of turnip accessions. “R” indicates the correlation index.

**Fig. S5** Correlation relationships between *FRIa* expression levels with flowering time (**A**) or altitude (**B**) among most of turnip accessions. “R” indicates the correlation index.

**Fig. S6** Correlation relationships between the expression levels of MADS-box genes and flowering time among different turnip accessions. “R” indicates the correlation index.

**Fig. S7** Correlation relationships between the expression levels of MADS-box genes and altitude among different turnip accessions. “R” indicates the correlation index.

**Fig. S8** Sequence analysis of the *FRIa* genotypes among turnip accessions. Representative DNA sequences of the *FRIa* genotypes obtained from the Sanger sequencing of the PCR fragments from genomic DNA.

**Fig. S9** Expression levels of *FRIa* in representative turnip accessions carrying each type of *FRIa* genotypes. Twenty-day-old seedlings were harvested for RT‒qPCR assays. Data are presented as mean ± *SD*, n = 3.

**Fig. S10** Identification of transgenic plants overexpressing the *FRIa* alleles. **A**. Expression level of *FRIa* in WT, *35S::GFP* and *FRIa1-OE~ FRIa5-OE* transgenic lines. Twelve-day-old seedlings were harvested for RT‒qPCR assays. Data are presented as mean ± *SD*, n = 3. **B**. Subcellular localization of FRIa-GFP fusion proteins in root tips of 10-day-old transgenic seedlings. Scar bar = 20 µm.

**Fig. S11** Functional validation of five *FRIa* alleles in regulating flowering time. **A**. Flowering times of WT, the plants transformed with empty vector (*GFP-OE*) and transgenic plants overexpressing of *FRIa* alleles (*FRIa1~5-OE*). Flowering time was expressed as days to bolting. Data are presented as the mean ± *SD*, n = 12. ***P < 0.001. **B**. *FLC* expression levels in the leaves of WT and transgenic plants. 12‐d‐old seedlings were harvested for RT‒qPCR assays. Data are presented as the mean ± *SD*, n = 3. “Dead” indicates the plants died before flowering.

**Fig. S12** Association analysis between SNPs and flowering time of turnip accessions carrying different SNP loci of *FRIa* alleles. The data were analyzed using Students’ *t*- test, ***P < 0.001; *P < 0.05; ns, not significant.

**Fig. S13** Expression levels of *FRIa* in WT and transgenic plants overexpressing *FRIa1* (*FRIa1-OE*) or mutated *FRIa1-OE*. Transgenic plants of *snp2-OE*, *snp5-OE*, *snp7-OE*, *snp8/9-OE*, *InDel2-OE*, *InDel3-OE* and *Hap1-OE* grown at 22°C under long-day conditions. Twelve-day-old seedlings were harvested for RT‒qPCR assays. Data are presented as mean ± *SD*, n = 3.

**Fig. S14** Subcellular localization of GFP-FRIa fusion proteins in root tips of transgenic seedlings overexpressing *FRIa1-OE* or mutated *FRIa1-OE*. Transgenic plants of *snp2-OE*, *snp5-OE*, *snp7-OE*, *snp8/9-OE*, *InDel2-OE* and *InDel3-OE* were observed. Scar bar = 20 µm.

**Fig. S15** Functional identification of the key SNP variation in *FRIa*. **A**. Flowering time of WT, transgenic *35S::GFP* plants (*GFP-OE*) and transgenic plants overexpressing *FRIa1-OE* or mutated *FRIa1-OE* (*snp2-OE*, *snp5-OE*, *snp8-OE*, *snp9/10-OE*, *InDel2-OE* and *InDel3-OE*). Flowering time was expressed as days to bolting. Data are presented as the mean ± *SD*, n = 12. ***P < 0.001. **B**. *FLC* expression levels in the leaves of WT and transgenic plants. Twelve-day-old seedlings were harvested for RT‒qPCR assays. Data are presented as the mean ± *SD*, n = 3.

**Fig. S16** Functional validation of snp8, snp9 and snp10 variation in *FRIa*. **A**. Representative phenotypes of WT, transgenic *35S::GFP* plants and transgenic plants overexpressing *FRIa1-OE* or mutated *FRIa1-OE* (*snp8-OE*, *snp9-OE*, and *snp10-OE*) grown at 22°C under long-day conditions. Scale bars = 2 cm. **B**. Subcellular localization of FRIa-GFP fusion proteins in root tips of ten-day-old transgenic seedlings. Scar bar = 100 µm. **C**. Expression level of *FRIa* in WT and transgenic plants. Twelve-day-old seedlings were harvested for RT‒qPCR assays. Data are presented as mean ± *SD*, n = 3. **D**. The number of rosette leaves of WT and transgenic plants. Data are presented as the mean ± SD, n = 12. ***P < 0.001.

**Fig. S17** Association analysis between SNPs of the *FRIa* alleles and altitudes of the turnip planting regions. The data were analyzed using Students’ *t*- test, ***P < 0.01; **P < 0.01; *P < 0.05; ns, not significant.

**Fig. S18** The potential structural changes of FRIa protein caused by the key four SNP sites at the amino acid level. **A**. The predicted protein structure of FRIa-Hap1(left), the four SNPs were then introduced into the protein (right). Alpha helix is displayed in red and the random coil is displayed in green. **B**-**E**. Analysis of the hydrophobicity (**B**), atomic charge (**C**), intermolecular interactions (**D**), and secondary structure (**E**) of amino acids after mutations at these four sites.

**Fig. S19** Multiple alignments of *FRIa* gene sequences with *FRI* homologues in the Brassicaceae family. The functional FRI sequence in *Arabidopsis thaliana*, *Arabidopsis lyrate*, *Thlaspi arvense*, *Capsella rubella*, *Brassica oleracea*, *Brassica juncea*, *Brassica napus*, *Brassica carinata* and ecotypes or accessions in these species were obtained for alignment. The red box represents the four SNP sites.
